# Characterization of functional trait diversity among Indian cultivated and weedy rice populations

**DOI:** 10.1038/srep24176

**Published:** 2016-04-13

**Authors:** M. Rathore, Raghwendra Singh, B. Kumar, B. S. Chauhan

**Affiliations:** 1 Indian Council of Agricultural Research-Directorate of Weed Research, Jabalpur, Madhya Pradesh, India; 2 The Centre for Plant Science, Queensland Alliance for Agriculture and Food Innovation (QAAFI), The University of Queensland, Toowoomba, Queensland, 4350, Australia

## Abstract

Weedy rice, a menace in rice growing areas globally, is biosimilar having attributes similar to cultivated and wild rice, and therefore is difficult to manage. A study was initiated to characterize the functional traits of 76 weedy rice populations and commonly grown rice cultivars from different agro-climatic zones for nine morphological, five physiological, and three phenological parameters in a field experiment under an augmented block design. Comparison between weedy and cultivated rice revealed a difference in duration (days) from panicle emergence to heading as the most variable trait and awn length as the least variable one, as evidenced from their coefficients of variation. The results of principal component analysis revealed the first three principal components to represent 47.3% of the total variation, which indicates an important role of transpiration, conductance, leaf-air temperature difference, days to panicle emergence, days to heading, flag leaf length, SPAD (soil-plant analysis development), grain weight, plant height, and panicle length to the diversity in weedy rice populations. The variations existing in weedy rice population are a major reason for its wider adaptability to varied environmental conditions and also a problem while trying to manage it.

Although rice (*Oryza sativa* L.) is the staple food for about 50% of the world’s population, more than half of the crop is produced and consumed in India and China^1^. As the most prominent crop in India, rice plays a vital role in the food security of the sub-continent and like any other crop, suffers various constraints to its production and productivity including pests, diseases, and weeds. Amongst these, weedy rice (*Oryza sativa* f. *spontanea*) is a menace infesting rice fields globally, but most severely in Asia, North America, and the Caribbean^2^. First documented in 1846^3^ in the United States of America, the weed has now spread to other rice areas in China, Thailand, Sri Lanka, Vietnam, Philippines and presently, India. Initial studies report an annual loss of more than $50 million in the USA due to weedy rice^4^. It now infests about 3 million ha land in China and has reduced total crop yield by 3.4 million M tonnes^5^. In India, an infestation of 5–60% in different states with 11.3 to 44.3% infestation in farmers’ fields has been assessed^6^. A yield reduction of 30–60% has been documented in rice agricultural fields of Kerela^7^ and 10–45% in Eastern Singhbhum of Jharkhand^8^. Though other crops like jute (*Corchurus capsularis*), maize (*Zea mays*), and soybean (*Glycine max*) are also affected by weedy rice^9^, it remains a major problem where direct seeding of rice has replaced conventional transplanting for paddy cultivation due to water scarcity and rising labor wages. In direct-seeded rice, weedy rice, which otherwise would be suppressed due to water logging at early stages, now experiences primarily aerobic conditions which favor its growth and development. It competes with cultivated rice for space, air, light, and nutrition thus impeding growth of rice crop. As on date, there is no herbicide that selectively kills weedy rice in a field of rice, so weedy rice is flourishing^8^. It can only be controlled through an integrated approach including mechanical, chemical, and agronomic methods of weed management which aim to reduce the weed seed bank^10^. Hand weeding is not possible because weedy rice cannot be differentiated at early growth stages.

Being a conspecific taxon of the AA genome complex of rice, weedy rice shares traits of both cultivated as well as wild rice types^8^. It usually flowers earlier than cultivated rice, has a pigmented caryopsis, pigmented hulls with or without awns and its grains shatter easily, thus enhancing the weed seed bank^11^. Increased nitrogen use efficiency and variable seed dormancy is also reported in weedy rice^12^,^13^.

Of the three hypotheses of the origin of weedy rice include (i) de-domestication of cultivated rice^14^, (ii) adaptation of wild rice^15^,^16^, and (iii) natural hybridization of cultivated and wild rice^17^, and/or amongst weedy rice or weedy and cultivated rice^15^; the latter is the most accepted^18^. As a natural hybrid, immense morphological variations are expected and reported in weedy rice across the globe^19^,^20^. Though reports of heavy infestations and damage to rice production in India are increasing^8^, very little work has been conducted on Indian weedy rice.

In India, weedy rice is usually confused with endemic wild rice because of very limited information about its traits and how they differ from wild rice. As a natural hybrid between cultivated and wild rice, weedy rice attributes vary depending on who their progenitors are. Studies on weedy rice from two different provinces of China revealed a low diversity amongst the weedy rice population themselves, but a close clustering with locally grown cultivars than with other cultivated and wild rice^21^. A high morpho-physiological resemblance between weedy and cultivated rice has been reported from Okayama Prefecture, Japan^22^. Thus, resemblance/diversity of weedy rice was found to vary with geographical location. India has six agro-climatic zones where different rice cultivars are grown. Weedy rice from these zones ought to vary in their functional traits, but yet no information is available in this context from the Indian subcontinent. It also becomes necessary to figure out how similar or dissimilar weedy rice is to/from cultivated/wild rice as this understanding may play an important role in developing weedy rice management strategies in future.

A study was initiated with weedy rice, collected from different agro-climatic zones of India and few popular rice cultivars with the following objectives- (i) to determine variations in functional traits amongst weedy rice populations, (ii) to determine association between weedy rice morphotypes and agro climatic zones, and (iii) to evaluate traits for ability to distinguish between cultivated and weedy rice.

As genetic variations exist in the weedy rice populations, this study on variability of weedy rice from different parts of India is expected to give direction for development of effective management strategies for this menace.

## Results

### Variation in quantitative traits

For the parameters assessed, immense variation was observed in the Coefficient of Variance, which ranged from 0 to 39% (Table 1). The reproductive parameters [grains per panicle (GPP), length–breadth (LB) ratio of grain, panicle length (PL), flag leaf length (FLL), awn length (AL), 100 seed weight (STW), days to panicle emergence (DPE), days to heading (D50PE), days taken from panicle emergence to heading (DDPE)] and the photosynthetic rate revealed maximum and minimum variations while vegetative [plant height (PH) and tiller number (TN)] and physiological parameters [difference in temperature of leaf and air (T_l_-T_a_), SPAD (soil-plant analysis development), conductance, and transpiration] revealed intermediate variations. The most variable trait recorded was DDPE followed by days to heading (50% panicle emergence) in plants per row (CV 39%), photosynthetic rate (CV 29%), and grains per panicle (CV 20%). The lowest variable trait was awn length (CV 0%) followed by days to heading (CV 5%), days to panicle emergence (CV 6%), and SPAD (CV 6%). Plant height and tiller number at 60 DAS, photosynthesis, conductance, transpiration and differences in temperature between leaf surface and air were traits with intermediate variations.

Comparative analysis amongst weedy, cultivated, and wild rice reveals that grains per panicle and LB ratio had higher maximum values in cultivated rice; awn length, seed test weight, and photosynthesis in wild rice and remaining in weedy rice. The minimum values for all traits were higher in wild rice except for grains per panicle, panicle length, seed test weight, conductance and transpiration which were higher in cultivated rice.

### Variations in qualitative plant traits

Ligules of studied cultivated and wild rice accessions were 2-cleft and green in color. Collar color was green and auricles were yellow-green in color. Weedy rice populations were largely similar to cultivated rice, but exceptions were observed as pink in 5 morphotypes) and black collared (in 2 morphotypes), pink (in 5 morphotypes) and black auricles (in 5 morphotypes) and pink (in 4 morphotypes) and black ligule color (in 2 morphotypes). The blade architecture of the flag leaf was largely erect in weedy and cultivated rice, while it was both erect (1) and semi-erect (1) in wild rice. A majority of the germplasm signified an open plant habit.

### Variations in qualitative grain parameters

No awns were present in the grains of cultivated rice and 23 populations of weedy rice. One wild rice strain had straw colored awns while the others had brown awns. Weedy rice populations’ revealed large variations in awn color viz. straw (31), brown (22), and black (15). While cultivated rice had straw colored hulls, one wild rice strain had black hulls. Weedy rice revealed a large variation in hull color viz. straw (28), brown (27) and black (21). Seventy weedy rice populations had red-brown colored grains and six displayed white grains. Wild rice had red-brown grains while all cultivated rice, except one, had white grains.

### Correlation analysis

Correlation among the physiological and agronomic variables of weedy rice and cultivated accessions reveals that the number of grains per panicle was positively correlated with panicle length (0.219), DPE (0.298), and D50PE (0.318) and weakly correlated with other variables (Table 2). The LB ratio was weakly correlated with other variables. The SPAD was correlated with plant height (−0.328), awn length (−0.351), and DPE (−0.339). T_l_–T_a_ had strong negative correlation with transpiration (−0.708) and photosynthesis (−0.363). The variables positively correlated with photosynthesis were conductance (0.542), tiller number (0.436), and transpiration (0.337). Conductance was strongly and positively correlated with transpiration (0.859) and negatively correlated with T_l_–T_a_ (−0.707). The moderately correlated variables with panicle length were flag leaf length (0.575) and plant height (0.358). Flag leaf length was positively correlated with plant height (0.479) and photosynthesis (0.203). Tiller number was negatively correlated with TW (−0.303). DPE was strongly positively correlated with D50PE (0.793). DDPE was positively correlated with tiller number (0.410). The results suggest that all the physiological variables had strong correlations as compared to morphological variations in grain parameters.

### Principal Component Analysis (PCA)

PCA was used to understand how physiological and morphological parameters contributed variability amongst the 76 weedy, two wild rice accessions, and 10 cultivated rice varieties. On the basis of scree plot, six principal components having Eigen values more than 1 were chosen. The data presented in Table 3 represents the proportion of total variance explained by each principal component, which was additive with each new component contributing less than the preceding one to the explained variance (Supplementary material S1 may be referred to). They are arranged in decreasing order of importance. PCA showed that first six PCs explained 18.9, 15.9, 12.5, 11.7, 9.8, and 6.7% of the variation respectively (Table 3). PC 1 was strongly correlated with transpiration (0.866), conductance (0.843), T_l_–T_a_ (−0.748), photosynthesis (0.573), and flag leaf length (0.424). The highly correlated variables with PC2 were SPAD (−0.651), DPE (0.340), D50PE (0.536), TW (−0.464), flag leaf length (0.435), and conductance (−0.401). PC3 was highly correlated with D50PE (−0.655), plant height (0.652), panicle length (0.579, flag leaf length (0.489), and DPE (−0.450). The variables which are strongly correlated with PC4 were tiller number (0.841), DDPE (0.703), photosynthetic rate (0.517), and DPE (−0.355). PC5 was highly correlated with grains per panicle (0.612), panicle length (0.534), SPAD (0.341), and DDPE (0.325), and strongly correlated variables with PC6 were awn length (0.647), LB ratio (0.386), and SPAD (−0.310). On the basis of PCA analysis it was visible that physiological variables add more variability amongst the accessions as compared to the physical variables.

### Phylogenetic relationship within weedy rice accessions and cultivated varieties

**Figure 1:**
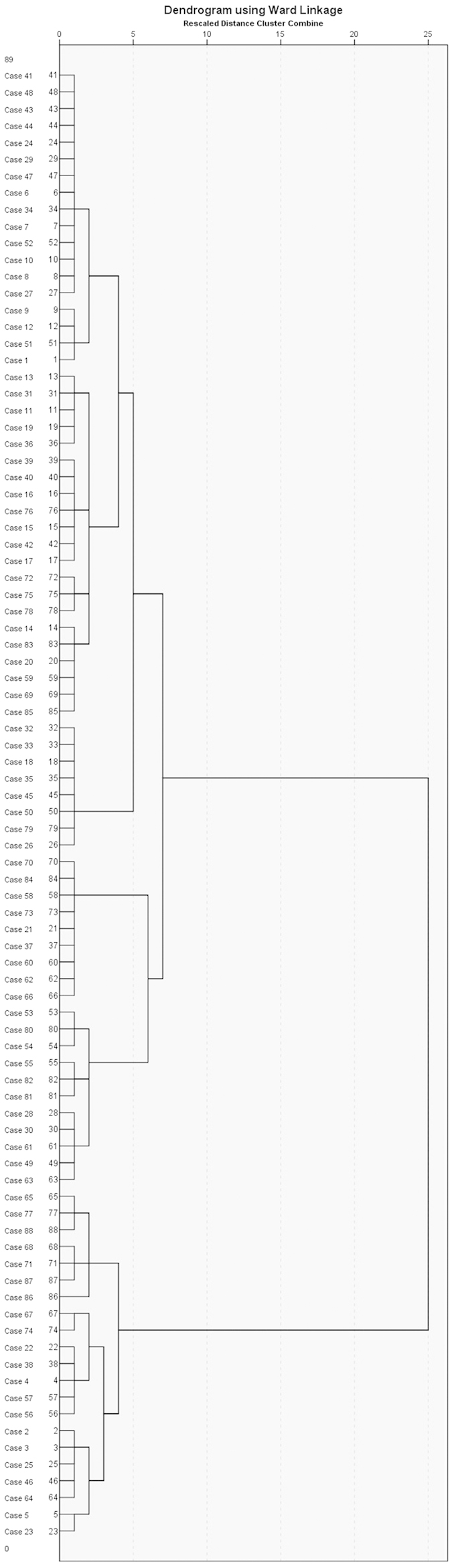
Dendrogram depicting phylogenetic relationship with in weedy rice morphotypes (numbers 11 to 86), cultivated (numbers 1 to 10) and wild rice varieties (number 87 and 88). G1 to G8 represent the groups which the rice samples fall into.

A dendrogram (Fig. 1) was generated using hierarchical cluster analysis using PC scores of different principle components, calculated on the basis of Eigen vectors of first six PCs for different variables for each accession. It reveals the germplasm studied to be sorted into eight groups classified from seven locations representing four agro-climatic zones of India (humid subtropical, tropical wet and dry, tropical wet, and semi-arid.). The germplasm clustered into groups were not similar on the basis of geographic location nor agro-climatic zones.

Information in Table 4 reveals that based on the studied morpho-physiological and phenological parameters, weedy rice clusters with cultivated rice (group 1, 2, and 3), with wild rice (group 8) and also remains as an independent group (groups 4, 5, 6, and 7). Weedy rice also clusters amongst itself, as evident by components of the independent groups. The dendrogram also reveals that groups 2, 3, and 8 are more distant from the other groups.

## Discussion

Results of the present investigation revealed large variations both amongst weedy rice populations and with cultivated rice. Plant height is an important parameter for competitive ability as taller weedy rice competes more efficiently for space and natural resources than the cultivated crop^23^. A majority (57%) of the weedy rice populations studied resulted in plant heights in the range of 75–95 cm while cultivated rice (70% population) resulted in heights of 82–92 cm. Only 19% of the weedy rice morphotypes had plant height in the range of 96–105 cm. This suggests that Indian weedy rice is not taller than cultivated rice. Thus, a similar range of plant heights would exacerbate camouflaging of weedy rice in a cultivated rice field and increase their chances of survival. The taller ones, though, would have a definite advantage of capturing more light over the neighboring plants in real field conditions^20^,^24^.

A rice tiller is a specialized grain-bearing-branch that is formed on the non-elongated basal internode that grows independent of the culm by means of its own adventitious roots. Tillering in rice is an important agronomic trait for grain production^25^, and profuse tillering is well known in weedy rice for its competitiveness^26^. The Indian weedy rice populations significantly varied in the tiller numbers they produced, but produced higher tiller numbers compared to cultivated rice.

Observations of the growth of the vegetative stage suggest that weedy rice has voracious growth due to profuse tillering and tall plants. These traits directly depend on the efficient utilization of available resources including sunlight and soil nitrogen (N). Chlorophyll content in leaves is an indirect measure of N status of the crop as a majority of leaf N is contained within the chlorophyll molecules^26^. The SPAD values from the chlorophyll meter provide instant relative chlorophyll status and crop N status in a non-destructive manner. However, no significant variation was observed in SPAD values between weedy rice and cultivated rice, indicating no inherent difference in chlorophyll content amongst them.

Photosynthetic efficiency (CO_2_ assimilation rate), transpiration, and conductance are other parameters regulating a plant’s growth and development. There were variations amongst weedy rice population and cultivated rice in terms of photosynthetic efficiency and conductance, but they were statistically non-significant. Transpiration is the process which, apart from cooling a plant, promotes water and nutrient absorption^28^ and is the physical driving force for cell enlargement. Though a majority of weedy rice populations had transpiration rates in a range (2.5–3.5 mmol H_2_O m^−2^ s^−1^) similar to cultivated rice, few populations revealed significantly higher values (3.6–4.4 mmol H_2_O m^−2^ s^−1^) compared to the other weedy rice populations. Variations were significant amongst them for transpiration rates, which may have exerted effect on leaf temperature, and hence significant differences in leaf-air temperature were observed. Stomatal conductance regulates gas flow and is known to be strongly correlated with leaf photosynthesis^29^,^30^. No significant variation was observed in stomatal conductance between weedy rice populations and cultivated rice.

It was observed that in weedy rice, by the end of its vegetative phase, physiological parameters were largely similar to cultivated rice, except for expected variations amongst the populations collected. These parameters, however, did not play a significant role in directly imparting competitiveness to weedy rice (under irrigated aerobic field conditions).

Panicle initiation followed by panicle emergence is the first sign of rice entering into the reproductive stage. The time (days) to the first panicle emergence revealed significant variations not only amongst weedy rice but also in cultivated rice. Similarly, time (days) to 50% heading revealed significant variations amongst weedy rice populations. Maximum variation was observed in days taken from panicle emergence to heading (DDPE). Though limited data is available for this trait in weedy rice, it is known that compared to cultivated rice, panicles emerge earlier, and mature asynchronously in weedy rice^26^ because they have attributes of both cultivated and wild rice, and wild rice is known to have asynchronous maturity in tiller emergence and panicle maturity^31^. As a result, variation in DDPE, a parameter reflecting heterogeneity in flowering, is expected. A majority of cultivated rice (60%) had a DDPE between 5–8 days while weedy rice populations (29%) had it between 3–5 days, indicating a shorter duration between panicle emergence and heading for weedy rice populations studied. The rapid emergence of panicles benefits weedy rice by allowing for earlier and easier exploitation of available natural resources in comparison to the field crop and thus, affecting rice yield^32^,^33^. Asynchronous flowering (as depicted by variations in DPE and DDPE) amongst weedy rice populations permits continuous maturing of seeds over a period of time. This enhances the probability of weedy rice to naturally cross pollinate with other morphotypes (populations) and cultivated rice, too. This, in turn, adds to the evolutionary complex of weedy rice in total.

Emergence of the flag leaf is also a characteristic of the reproductive stage and a quantitative descriptor for rice germplasm characterization along with panicle length. Significant variations were found between weedy populations for these traits.

Like cultivated rice, 86% of weedy rice populations had an LB ratio in range of 2.0–3.0. Variations in the LB ratio, and hence grain size, were expected as weedy rice largely evolve by natural hybridization amongst and/or between cultivated/wild and weedy rice morphotypes.

A majority of the grains of weedy rice populations had awns (71%) though they varied significantly in length ranging from 0 to 7.8 mm. Domesticated rice lines usually have short awns, while wild relatives have longer awns^34^. Natural hybrids between cultivated and wild rice, the weedy populations, thus, may have varied awn lengths as evident from this study.

While strong correlations amongst physiological parameters are expected, correlations between panicle length and flag leaf length and, in turn, with plant height and photosynthetic rate were also observed during the study. Weedy rice plants with increased height will have more access to sunlight and air and hence will photosynthesize more efficiently (keeping all other variables required for photosynthesis as constant). During the reproductive stage, the flag leaf is an important source of photosynthetic energy and grain filling and hence affects panicle development^35^ (here panicle length).

Grains per panicle showed a positive correlation to panicle length, days to panicle emergence and days to heading. A weedy rice strain entering the reproductive stage relatively earlier (as evident by panicle emergence and D50PE) would, again, utilize the available natural resources more efficiently than its conventional counterpart (the field crop). This would, in turn, aid in the development of panicle and grains. A recent study found similar results, wherein days to heading in rice had a positive correlation with grain yield^36^.

Though several traits revealed non-significant variations amongst weedy rice and with cultivated rice based on a simple statistical analysis, they were important for adding to and for explaining the existing variability amongst the populations based on Principal Component Analysis, for example, photosynthetic rate that added more variability amongst the populations as compared to the agronomic values. In short, each trait studied had an impact on the variability of weed rice populations. But though the diversity was large, the populations of Indian weedy rice did not distribute themselves among agro-climatic regions. Similar results have been found in Sri Lankan weedy rice^19^. This indicates that the agronomic and physiological traits were not associated to a particular region. There may be various reasons for this response, but a major reason is the seed traffic of cultivated rice across agro-climatic zones. Grains of cultivated rice are usually preserved from the harvest as seed for the next season and frequently exchanged amongst farmers. Weedy rice populations contaminate seeds, and such seeds thus spread and contaminate rice growing areas of the other agro-climatic regions. They can also easily cross hybridize with cultivars/other weedy rice morphotypes growing there and generate a varied complex or hybrid swarm. Also, rice cultivation patterns and systems vary across the agro-climatic regions due to precipitation patterns and prevailing socio-economic situation in India^37^.

This study documents the existing variations amongst the functional traits of Indian weedy rice and suggests that they cannot be used to differentiate it from cultivated rice. This implies that strategies for management of weedy rice cannot be developed using the existing diversity in functional trait parameters. But because phenotypic variations are a reflection of underlying genetic diversity, differences at molecular level may be studied for finding potential differences that may be targeted for developing management strategies. Also, as statistical analysis based on functional traits reveals that weedy rice morphotypes do not distribute based on agro-climatic zones, molecular studies can aid in revealing and understanding any underlying population structure based on genetic similarity.

This study documents existing variations amongst functional traits of Indian weedy rice and reveals phenotypic and seed mimicry with cultivated rice. Chronological mimicry is absent as weedy rice panicles emerge and mature asynchronously and earlier than cultivated rice. Also, high seed shattering is documented in weedy rice. This trait, however, cannot be used for developing management strategies because by the time panicles emerge in weedy rice, the damage has already been done. This implies that strategies for management of weedy rice cannot be developed using the existing diversity in functional trait parameters. It is understood that phenotypic variations are a reflection of underlying genetic diversity, hence differences at molecular level may be studied and targeted for developing management strategies. Development of Clearfield rice (rice resistant to the imidazolinone group of herbicides that permits selective chemical control of weedy rice) and its use to control weedy rice in Malaysia is one such example^38^,^39^,^40^. Hence, diversity in functional traits is not sufficient to devise strategies for management of weedy rice and interventions at the molecular level are desirable in this context.

## Material and Methods

### Seed collection

Mature seeds of weedy rice were collected from farmers’ fields across different agro-climatic zones of India during rice growing season(s) in 2011. Exact locations of morphotypes collected (Supplementary Table S2)^41^ were recorded with the help of a Global Positioning System (GPS) (Garmin Oregon 550). Two accessions of wild rice were collected from Central Rice Research Institute, Cuttack, India. Ten commonly grown rice varieties from different states (Madhya Pradesh, Uttar Pradesh, Bihar, Jharkhand, and Chhattisgarh) were used.

### Field experiment

During the summer season of 2012 (June–November), collected weedy rice seeds were sown in a field with no previous history of weedy rice. Sowing was done by direct seeding in an augmented block design. To maintain real field conditions, seeds were not given any treatment before sowing and seeds intact with awn were sown.

The field was divided into three blocks. Two of the blocks had 26 weedy rice morphotypes each along with 10 cultivated rice cultivars as the check. The third block had only 24 weedy rice morphotypes and two wild rice lines along with 10 rice cultivars as the check. Each morphotype was present in a single row of 10 m length at a plant to plant spacing of 50 cm.

The field was irrigated when needed. Bispyribac-sodium 25 g a.i. ha^−1^ was applied as post-emergent to manage narrow and broad leaved weeds. The herbicide was applied using a flat fan nozzle fitted with a backpack sprayer. Further flushes of *Echinochloa colona*, *Cyperus iria*, and *Cyperus tenuispica* were controlled by hand weeding.

### Morphological evaluation

All weedy rice populations and rice cultivars were evaluated 60 days after sowing (DAS) and at harvest. A total of 11 vegetative and reproductive parameters (Table 5) were assessed using the IRRI Standard Evaluation system for rice^42^. The traits assessed were expressed on a continuous scale. Seeds were broadly categorized as awned or awnless. Total number of grains per panicle (GPP) was estimated by counting the number of pedicels per panicle assuming that a seed was formed at each pedicel^43^. Traits evaluated on a non-continuous scale include culm color, collar color, presence of ligule, and color of auricle (the discontinuous variables).

### Physiological evaluation

Chlorophyll content was determined using SPAD −502 (Soil-Plant Analysis Development) chlorophyll meter (Minolta Co., Ltd, Osaka, Japan). Photosynthetic rate, stomatal conductance, transpiration rate, leaf and air temperature differences were determined using LI-COR (LI-6400XT Portable Photosynthesis System, Lincoln, Nebraska, USA). The measurements were taken randomly on the abaxial surface of three leaves for each population at 60 days after sowing.

### Statistical analyses

The differences in mean values between quantitative traits of different accessions were estimated through analysis of variance (ANOVA) adopting General Linear Model (GLM) using SAS software. The quantitative descriptors were subjected to hierarchical cluster analysis and associated dendrogram using SAS 9.3, SAS Institute. Principal Component Analysis (PCA) based on Eigen values for step-wise observations among different weedy rice accessions was also analyzed to determine relationships among the weedy rice accessions collected from different agro-climatic zones and with cultivated rice. Scatter plots for Principal components analyzed are given in supplementary material S1.

## Additional Information

**How to cite this article**: Rathore, M. *et al.* Characterization of functional trait diversity among Indian cultivated and weedy rice populations. *Sci. Rep.* **6**, 24176; doi: 10.1038/srep24176 (2016).

## Supplementary Material

Supplementary Information

## Figures and Tables

**Table 1:** Trait descriptions in study.

Trait	Unit	Maximum Value		Minimum Value	Mean Value	SD	Coefficient of Variation (%)				
GPP	Number	WR	333.3	43.3	143.8	61.283	20				
		CR	401.6	104.1	212.2	96.963					
LB ratio	–	WR	5.01	2.01	2.80	0.673	12.29				
		CR	6.5	2.67	3.58	1.173					
PL	Number	WR	31.03	16.4	24.8	3.529	10.3				
		CR	29.06	22.05	24.7	2.491					
FLL	cm	WR	54.0	5.67	34.06	7.492	9.09				
		CR	32.82	24.03	28.84	2.452					
PH	cm	WR	144.98	56.19	94.89	16.952	9.92				
		CR	92.43	72.4	85.56	5.893					
AL	cm	WR	7.8	0	2.9	0.673	0				
		CR	0	0	0	0					
TN	number	WR	137.1	12.3	43.15	21.265	16.73				
		CR	39.53	19.5	27.1	5.468					
STW	gram	WR	2.93	1.59	2.3	0.348	14.43				
		CR	2.86	2.04	2.60	0.279					
T_l_–T_a_	^0^C	WR	−3.16	−0.83	−2.03	−0.554	−12.31				
		CR	−2.79	−1.54	−2.29	−0.340					
SPAD	–	WR	46.2	31.2	38.6	2.750	6.24				
		CR	43.8	39.68	41.6	1.520					
Photosynthesis	μmole CO_2_ m^−2^_leaf area_ s ^−1^	WR	16.89	2.03	10.2	4.082	29.12				
		CR	12.65	7.3	18.43	1.934					
Conductance	mol H_2_O m^−2^ s ^−1^	WR	0.0877	0.031	0.061	0.0115	15.24				
		CR	0.070	0.0525	0.061	0.007					
Transpiration	mMol H_2_O m^−2^ s ^−1^	WR	4.43	1.21	3.00	0.581	8.46				
		CR	3.5	2.56	3.02	0.417					
DTPE	days	WR	97.9	52.9	76.9	11.946	6.13				
		CR	88.67	60.3	76.4	13.872					
DT50PE	days	WR	107.3	59.8	83.9	11.688	5.01				
		CR	103	67	82.1	12.537					
DDPE	days	WR	37.47	1.46	7.27	6.904	39.0				
		CR	9.67	4.67	7.15	1.920					

GPP: grains per panicle, LB ratio: length breadth ratio of grain, PL: panicle length, FLL: flag leaf length, PH: plant height, AL: awn length, TN: tiller number, STW: seed test weight, Tl–Ta: difference in leaf temperature and air temperature, DTPE: days to panicle emergence, DT50PE: days to 50% panicle emergence, DDPE: difference between DTPE and DT50PE, WR: weedy rice, CR: cultivated rice, cm: centimeter, ^°^C: degree centigrade, μ: micro, m: milli SD: standard deviation.

**Table 2:** Correlation matrix of different physiological and agronomic variables.

	Correlation Matrix																
		GPP	LB ratio	SPAD	Tl–Ta	Photo-synthesis	conductance	PL	FLL	PH	AL	TN	Transpiration	TW	DTPE	DT50PE	DDPE
Co−relation	GPP	1.000	0.176	0.028	−0.147	−0.013	0.031	0.219	0.067	0.086	−0.122	−0.108	0.115	−0.021	0.298	0.318	0.074
	LB ratio		1.00	0.194	0.014	−0.141	−0.079	0.249	−0.076	−0.114	−0.074	−0.095	−0.065	0.045	−0.193	−0.066	0.220
	SPAD			1.000	−0.047	0.232	0.139	−0.071	−0.188	−0.328	−0.351	−0.145	−0.118	0.164	−0.339	−0.233	0.126
	Tl−Ta				1.000	−0.363	−0.707	0.044	−0.047	0.099	0.178	0.088	−0.708	−0.083	−0.126	−0.156	−0.015
	photosynthesis					1.000	0.542	0.001	0.203	0.251	−0.050	0.436	0.337	0.107	−0.180	−0.031	0.204
	conductance						1.000	0.016	0.129	0.095	−0.021	−0.038	0.859	0.163	0.026	0.055	0.056
	PL							1.000	0.575	0.358	0.172	−0.037	0.046	−0.043	0.012	−0.057	−0.109
	FLL								1.000	0.479	0.057	0.143	0.196	−0.178	0.173	0.116	−0.112
	PH								.	1.000	0.273	0.078	0.124	0.126	0.081	−0.070	−0.182
	AL										1.000	0.131	0.017	−0.124	0.121	0.120	0.078
	TN											1.000	−0.059	−0.303	−0.053	0.180	0.410
	transpiration												1.000	0.128	0.189	0.152	−0.057
	TW													1.000	−0.234	−0.285	−0.130
	DTPE														1.000	0.793	−0.291
	DT50PE															1.000	0.281
	DDPE																1.000

**Table 3:** Total variance explained through Principal Component Analysis of first 6 Principal components.

Component	Total	% of Variance	Cumulative %
1	3.02	18.9	18.9
2	2.55	15.9	34.8
3	1.99	12.5	47.2
4	1.87	11.7	58.9
5	1.57	9.8	68.7
6	1.07	6.7	75.4

**Table 4:** Details of group components of dendrogram.

Group	Contributing Germplasm		Agro-climatic zone
	Number	Identity	
G1	18	Weedy (12) and cultivated (6)rice	•Humid subtropical
G2	7	Weedy (4) and cultivated (3) rice	•Humid subtropical
G3	7	Weedy (6) and cultivated (1)rice	•Humid subtropical
			•Semi-arid
G4	21	Weedy rice	•Humid subtropical
			•Tropical wet and dry
			•Tropical wet
G5	8	Weedy rice	•Humid subtropical
G6	9	Weedy rice	•Humid subtropical
			Semi-arid
G7	11	Weedy rice	•Humid subtropical
			•Semi-arid
G8	7	Weedy(5) and wild rice(2)	•Humid subtropical
			•Tropical wet

G: Group.

**Table 5:** Traits assessed for morphological characterization of Indian weedy rice and method of their measurement.

Trait	Abbreviated Code	Method of trait measurement
Plant height	PLH	Height from base to tip of plant 60 days after seed germination
Tiller no	TN	Number of tillers 60 days after seed germination
Days to panicle emergence (a)	DPE	Days from seed germination to panicle emergence
Days to heading (b)	D50PE	Days from seed germination to 50% panicle emergence
Days taken from panicle emergence to heading	DDPE	Difference between (a) and (b)
Flag leaf length	FLL	Length from base o tip of flag leaf
Panicle length	PL	Length from base to tip of panicle
Grains per panicle	GPP	Number of pedicels per panicle
Awn length	AL	Length from tip of grain/base of awn to tip of awn
Length- breadth ratio of grain	LBR	Ratio of length to width of grain in mm
100-seed weight	STW	Weight of 100 well developed seeds
