# Supplementary material for: Characterization of functional trait diversity among Indian cultivated and weedy rice populations
Source: Sci Rep. 2016 Apr 13;6:24176. doi: 10.1038/srep24176 (PMC4829852; doi:10.1038/srep24176)
Supplement: Supplementary Information [file srep24176-s1.doc]

Characterization of functional trait diversity among Indian cultivated and weedy rice populations
 Authors: Rathore M.^*^, Singh Raghwendra, Kumar B. and Chauhan, B.S^*^.

Principal Components Analysis Supplementary material S1

The PRINCOMP Procedure

Observations 88

Variables 16

| Simple Statistics | | | | | | | | |
| --- | --- | --- | --- | --- | --- | --- | --- | --- |
|  | grainsperpanicle | lbratio | SPAD | Tl-Ta | photosyn | conductance | paniclelength | FLLength |
| Mean | 148.2110530 | 2.884280303 | 39.13431818 | -2.076977273 | 10.05994318 | 0.0618409091 | 24.68996495 | 33.10376983 |
| StD | 70.5261042 | 0.775417205 | 3.08004116 | 0.526486085 | 3.27297062 | 0.0109648853 | 3.25810385 | 7.97326080 |

| Simple Statistics | | | | | | | | |
| --- | --- | --- | --- | --- | --- | --- | --- | --- |
|  | plantheight | awnlength | tillerno | transpiration | TW | DTPI | DTPE50p | DiffPIPE |
| Mean | 94.11954545 | 2.494318182 | 41.16800000 | 3.008799242 | 2.309079545 | 76.42121215 | 83.59318182 | 7.412121242 |
| StD | 16.17984423 | 2.293073426 | 20.11681201 | 0.568000480 | 0.357113710 | 11.81166402 | 11.70619702 | 6.444129036 |

| Correlation Matrix | | | | | | | | |
| --- | --- | --- | --- | --- | --- | --- | --- | --- |
|  | grainsperpanicle | lbratio | SPAD | Tl-Ta | photosyn | conductance | paniclelength | FLLength |
| grainsperpanicle | 1.0000 | 0.1759 | 0.0278 | -.1473 | -.0133 | 0.0312 | 0.2185 | 0.0674 |
| lbratio | 0.1759 | 1.0000 | 0.1940 | 0.0136 | -.1406 | -.0789 | 0.2485 | -.0755 |
| SPAD | 0.0278 | 0.1940 | 1.0000 | -.0470 | 0.2317 | 0.1387 | -.0713 | -.1880 |
| Tl-Ta | -.1473 | 0.0136 | -.0470 | 1.0000 | -.3634 | -.7069 | 0.0438 | -.0474 |
| photosyn | -.0133 | -.1406 | 0.2317 | -.3634 | 1.0000 | 0.5424 | 0.0013 | 0.2026 |
| conductance | 0.0312 | -.0789 | 0.1387 | -.7069 | 0.5424 | 1.0000 | 0.0157 | 0.1285 |
| paniclelength | 0.2185 | 0.2485 | -.0713 | 0.0438 | 0.0013 | 0.0157 | 1.0000 | 0.5747 |
| FLLength | 0.0674 | -.0755 | -.1880 | -.0474 | 0.2026 | 0.1285 | 0.5747 | 1.0000 |
| plantheight | 0.0865 | -.1142 | -.3278 | 0.0993 | 0.2512 | 0.0954 | 0.3582 | 0.4789 |
| awnlength | -.1215 | -.0743 | -.3506 | 0.1779 | -.0498 | -.0212 | 0.1718 | 0.0566 |
| tillerno | -.1075 | -.0952 | -.1449 | 0.0880 | 0.4361 | -.0378 | -.0367 | 0.1428 |
| transpiration | 0.1153 | -.0652 | -.1176 | -.7082 | 0.3373 | 0.8587 | 0.0464 | 0.1964 |
| TW | -.0208 | 0.0454 | 0.1637 | -.0831 | 0.1073 | 0.1627 | -.0426 | -.1784 |
| DTPI | 0.2976 | -.1929 | -.3385 | -.1257 | -.1798 | 0.0255 | 0.0120 | 0.1725 |
| DTPE50p | 0.3183 | -.0659 | -.2329 | -.1557 | -.0305 | 0.0550 | -.0566 | 0.1157 |
| DiffPIPE | 0.0742 | 0.2195 | 0.1257 | -.0153 | 0.2039 | 0.0557 | -.1086 | -.1117 |

Generated by the SAS System ('Local', W32_7HOME) on 07 July 2014 at 12:29:35 PM

07:37 Tuesday, February 02, 2016 2

Principal Components Analysis

The PRINCOMP Procedure

| Correlation Matrix | | | | | | | | |
| --- | --- | --- | --- | --- | --- | --- | --- | --- |
|  | plantheight | awnlength | tillerno | transpiration | TW | DTPI | DTPE50p | DiffPIPE |
| grainsperpanicle | 0.0865 | -.1215 | -.1075 | 0.1153 | -.0208 | 0.2976 | 0.3183 | 0.0742 |
| lbratio | -.1142 | -.0743 | -.0952 | -.0652 | 0.0454 | -.1929 | -.0659 | 0.2195 |
| SPAD | -.3278 | -.3506 | -.1449 | -.1176 | 0.1637 | -.3385 | -.2329 | 0.1257 |
| Tl-Ta | 0.0993 | 0.1779 | 0.0880 | -.7082 | -.0831 | -.1257 | -.1557 | -.0153 |
| photosyn | 0.2512 | -.0498 | 0.4361 | 0.3373 | 0.1073 | -.1798 | -.0305 | 0.2039 |
| conductance | 0.0954 | -.0212 | -.0378 | 0.8587 | 0.1627 | 0.0255 | 0.0550 | 0.0557 |
| paniclelength | 0.3582 | 0.1718 | -.0367 | 0.0464 | -.0426 | 0.0120 | -.0566 | -.1086 |
| FLLength | 0.4789 | 0.0566 | 0.1428 | 0.1964 | -.1784 | 0.1725 | 0.1157 | -.1117 |
| plantheight | 1.0000 | 0.2733 | 0.0776 | 0.1241 | 0.1260 | 0.0814 | -.0697 | -.1824 |
| awnlength | 0.2733 | 1.0000 | 0.1312 | 0.0166 | -.1242 | 0.1213 | 0.1203 | 0.0776 |
| tillerno | 0.0776 | 0.1312 | 1.0000 | -.0589 | -.3025 | -.0526 | 0.1799 | 0.4101 |
| transpiration | 0.1241 | 0.0166 | -.0589 | 1.0000 | 0.1280 | 0.1888 | 0.1520 | -.0571 |
| TW | 0.1260 | -.1242 | -.3025 | 0.1280 | 1.0000 | -.2337 | -.2849 | -.1302 |
| DTPI | 0.0814 | 0.1213 | -.0526 | 0.1888 | -.2337 | 1.0000 | 0.7926 | -.2913 |
| DTPE50p | -.0697 | 0.1203 | 0.1799 | 0.1520 | -.2849 | 0.7926 | 1.0000 | 0.2812 |
| DiffPIPE | -.1824 | 0.0776 | 0.4101 | -.0571 | -.1302 | -.2913 | 0.2812 | 1.0000 |

| Eigenvalues of the Correlation Matrix | | | | |
| --- | --- | --- | --- | --- |
|  | Eigenvalue | Difference | Proportion | Cumulative |
| 1 | 3.01823562 | 0.47204696 | 0.1886 | 0.1886 |
| 2 | 2.54618865 | 0.55338250 | 0.1591 | 0.3478 |
| 3 | 1.99280615 | 0.12350123 | 0.1246 | 0.4723 |
| 4 | 1.86930492 | 0.30433154 | 0.1168 | 0.5892 |
| 5 | 1.56497339 | 0.49777320 | 0.0978 | 0.6870 |
| 6 | 1.06720018 | 0.14383642 | 0.0667 | 0.7537 |
| 7 | 0.92336376 | 0.26044810 | 0.0577 | 0.8114 |
| 8 | 0.66291566 | 0.07995255 | 0.0414 | 0.8528 |
| 9 | 0.58296311 | 0.07023927 | 0.0364 | 0.8892 |
| 10 | 0.51272384 | 0.09075363 | 0.0320 | 0.9213 |
| 11 | 0.42197022 | 0.12659159 | 0.0264 | 0.9477 |
| 12 | 0.29537862 | 0.05463973 | 0.0185 | 0.9661 |
| 13 | 0.24073890 | 0.04652721 | 0.0150 | 0.9812 |

Generated by the SAS System ('Local', W32_7HOME) on 07 July 2014 at 12:29:35 PM


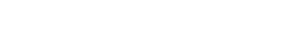

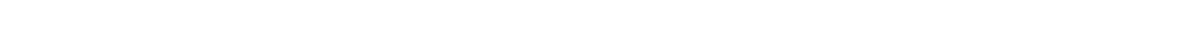
07:37 Tuesday, February 02, 2016 3

Principal Components Analysis

The PRINCOMP Procedure

| Eigenvalues of the Correlation Matrix | | | | |
| --- | --- | --- | --- | --- |
|  | Eigenvalue | Difference | Proportion | Cumulative |
| 14 | 0.19421169 | 0.11921521 | 0.0121 | 0.9933 |
| 15 | 0.07499647 | 0.04296767 | 0.0047 | 0.9980 |
| 16 | 0.03202880 |  | 0.0020 | 1.0000 |

| Eigenvectors | | | | | | | | | |
| --- | --- | --- | --- | --- | --- | --- | --- | --- | --- |
|  | PRIN1 | PRIN2 | PRIN3 | PRIN4 | PRIN5 | PRIN6 | PRIN7 | PRIN8 | PRIN9 |
| grainsperpanicle | 0.139286 | 0.119293 | -.148248 | -.166457 | 0.488846 | -.081661 | 0.439064 | -.088896 | -.615234 |
| lbratio | -.090306 | -.102792 | 0.023277 | -.006543 | 0.582629 | 0.373863 | -.152607 | -.210755 | 0.287452 |
| SPAD | -.074365 | -.407883 | -.035045 | 0.033720 | 0.272769 | -.300043 | 0.028320 | 0.665173 | 0.053297 |
| Tl-Ta | -.430778 | 0.220648 | 0.163971 | 0.112721 | -.012201 | -.051890 | 0.194260 | 0.168021 | 0.081128 |
| photosyn | 0.329756 | -.205888 | 0.128831 | 0.377869 | -.049677 | -.230679 | 0.239049 | 0.136243 | -.017396 |
| conductance | 0.485367 | -.251425 | -.001231 | -.011444 | -.062656 | 0.126472 | -.108461 | 0.138202 | -.029982 |
| paniclelength | 0.110789 | 0.209119 | 0.410336 | -.043492 | 0.426698 | 0.018148 | -.205699 | 0.158724 | 0.029512 |
| FLLength | 0.243946 | 0.272707 | 0.346247 | 0.068503 | 0.165277 | -.319078 | -.220974 | -.004214 | 0.264436 |
| plantheight | 0.182624 | 0.237918 | 0.461653 | 0.028883 | -.054909 | 0.019535 | 0.379091 | -.101640 | -.050831 |
| awnlength | 0.028407 | 0.284695 | 0.123681 | 0.156979 | -.180587 | 0.626555 | 0.000602 | 0.526785 | -.197871 |
| tillerno | 0.065096 | 0.117656 | -.046495 | 0.614878 | -.063869 | -.139828 | 0.055032 | -.233642 | 0.048371 |
| transpiration | 0.498434 | -.120945 | -.030059 | -.122081 | -.073727 | 0.200323 | -.154349 | -.115054 | -.028495 |
| TW | 0.021531 | -.290553 | 0.210120 | -.239340 | -.059347 | 0.205509 | 0.602323 | -.055306 | 0.469127 |
| DTPI | 0.195919 | 0.407187 | -.318939 | -.259447 | -.040283 | -.129777 | 0.099182 | 0.173327 | 0.241569 |
| DTPE50p | 0.198878 | 0.336206 | -.463693 | 0.041704 | 0.116818 | -.004742 | 0.164459 | 0.151649 | 0.358982 |
| DiffPIPE | 0.002911 | -.084037 | -.228330 | 0.513922 | 0.259436 | 0.278851 | 0.122906 | -.048624 | 0.072426 |

| Eigenvectors | | | | | | | |
| --- | --- | --- | --- | --- | --- | --- | --- |
|  | PRIN10 | PRIN11 | PRIN12 | PRIN13 | PRIN14 | PRIN15 | PRIN16 |
| grainsperpanicle | -.038737 | -.115739 | 0.136424 | 0.223132 | -.033013 | -.078298 | -.036555 |
| lbratio | 0.535244 | 0.182484 | 0.002175 | 0.147978 | -.085752 | -.045374 | -.005227 |
| SPAD | 0.090118 | 0.119842 | 0.104702 | 0.034152 | 0.405719 | 0.126356 | 0.004916 |
| Tl-Ta | 0.009649 | 0.150723 | 0.673249 | -.124796 | -.398300 | 0.020219 | -.007812 |
| photosyn | 0.292297 | -.041080 | -.278492 | 0.028104 | -.588129 | 0.199273 | 0.081982 |
| conductance | -.013086 | 0.124462 | 0.289551 | -.203094 | -.116332 | -.693928 | -.116489 |
| paniclelength | -.145513 | -.469329 | -.079578 | -.518013 | -.040351 | 0.069377 | 0.019872 |

Generated by the SAS System ('Local', W32_7HOME) on 07 July 2014 at 12:29:35 PM

**Supplementary table S2**

**Characterization of functional trait diversity among Indian cultivated and weedy rice populations**

**Authors:** Rathore M**.**^*^, Singh Raghwendra, Kumar B. and Chauhan, B.S^*^.

**Table S2.** Indian weedy rice populations collected from different agro-climatic zones^43^

| **S. no.** | **Population code used in dendogram** | **Area** | **latitude** | **Longitude** | **Agro-climatic zone** |
| --- | --- | --- | --- | --- | --- |
| 1 | 11 | Jabalpur | no data* | no data* | Humid subtropical |
| 2 | 12 | Jabalpur | no data* | no data* | Humid subtropical |
| 3 | 13 | Jabalpur | no data* | no data* | Humid subtropical |
| 4 | 14 | Jabalpur | no data* | no data* | Humid subtropical |
| 5 | 15 | Jabalpur | no data* | no data* | Humid subtropical |
| 6 | 16 | Jabalpur | no data* | no data* | Humid subtropical |
| 7 | 17 | Jabalpur | no data* | no data* | Humid subtropical |
| 8 | 18 | Jabalpur | no data* | no data* | Humid subtropical |
| 9 | 19 | Jabalpur | no data* | no data* | Humid subtropical |
| 10 | 20 | Jabalpur | no data* | no data* | Humid subtropical |
| 11 | 21 | Jabalpur | no data* | no data* | Humid subtropical |
| 12 | 22 | Jabalpur | 23^0^13’57.8” N | 79^0^58’08.7”E | Humid subtropical |
| 13 | 23 | Jabalpur | 23^0^13’49.6” N | 79^0^58’18.8”E | Humid subtropical |
| 14 | 24 | Jabalpur | 23^0^13’50.9” N | 79^0^58’14.6”E | Humid subtropical |
| 15 | 25 | Jabalpur | 23^0^13’48.8” N | 79^0^58’10.4”E | Humid subtropical |
| 16 | 26 | Jabalpur | 23^0^13’51.7” N | 79^0^58’18.6”E | Humid subtropical |
| 17 | 27 | Jabalpur | 23^0^13’48.4” N | 79^0^58’18.5”E | Humid subtropical |
| 18 | 28 | Jabalpur | 23^0^13’56.8” N | 79^0^58’04.9”E | Humid subtropical |
| 19 | 29 | Jabalpur | 23^0^13’53.3” N | 79^0^58’05.4”E | Humid subtropical |
| 20 | 30 | Jabalpur | 23^0^13’51.2” N | 79^0^58’05.9”E | Humid subtropical |
| 21 | 31 | Jabalpur | 23^0^13’51.0” N | 79^0^58’08.2”E | Humid subtropical |
| 22 | 32 | Jabalpur | 23^0^13’50.2” N | 79^0^58’14.5”E | Humid subtropical |
| 23 | 33 | Jabalpur | 23^0^13’46.8” N | 79^0^58’14.3”E | Humid subtropical |
| 24 | 34 | Jabalpur | 23^0^13’49.8” N | 79^0^58’17.3”E | Humid subtropical |
| 25 | 35 | Jabalpur | 23^0^13’45.6” N | 79^0^58’17.9”E | Humid subtropical |
| 26 | 36 | Jabalpur | 23^0^13’44.9” N | 79^0^58’18.7”E | Humid subtropical |
| 27 | 37 | Jabalpur | 23^0^13’49.1” N | 79^0^58’27.8”E | Humid subtropical |
| 28 | 38 | Jabalpur | 23^0^13’49.4” N | 79^0^58’18.8”E | Humid subtropical |
| 29 | 39 | Jabalpur | 23^0^13’46.2” N | 79^0^58’18.7”E | Humid subtropical |
| 30 | 40 | Jabalpur | 23^0^13’48.1” N | 79^0^58’18.8”E | Humid subtropical |
| 31 | 41 | Panagar | 23^0^16’28.8” N | 79^0^59’52.0”E | Humid subtropical |
| 32 | 42 | Panagar | 23^0^16’01.0” N | 80^0^00’07.1”E | Humid subtropical |
| 33 | 43 | Panagar | 23^0^16’08.0” N | 80^0^00’07.1”E | Humid subtropical |
| 34 | 44 | Panagar | 23^0^16’04.0” N | 79^0^59’55.6”E | Humid subtropical |
| 35 | 45 | Panagar | 23^0^16’04.8” N | 79^0^59’52.5”E | Humid subtropical |
| 36 | 46 | Panagar | 23^0^16’04.4” N | 79^0^59’53.6”E | Humid subtropical |
| 37 | 47 | Panagar | 23^0^16’02.1” N | 80^0^00’08.5”E | Humid subtropical |
| 38 | 48 | Mehgawa | 23^0^19’05.9” N | 80^0^02’16.6”E | Humid subtropical |
| 39 | 49 | Mehgawa | 23^0^19’02.0” N | 80^0^02’21.5”E | Humid subtropical |
| 40 | 50 | Mehgawa | 23^0^19’02.6” N | 80^0^02’22.2”E | Humid subtropical |
| 41 | 51 | Mehgawa | 23^0^19’01.5” N | 80^0^01’50.9”E | Humid subtropical |
| 42 | 52 | Ranchi | 23^0^14’51.6” N | 85^0^16’53.68”E | Humid subtropical |
| 43 | 53 | Ranchi | 23^0^14’33.04” N | 85^0^17’12.53”E | Humid subtropical |
| 44 | 54 | Raipur | 21^0^13’32.63” N | 81^0^41 01.81”E | Humid subtropical |
|  |  |  |  |  |  |
| 45 | 55 | Bhind, Gwalior | 26^0^ 34’28.87” N | 78^0^45’ 16.51”E | Semi-arid |
| 46 | 56 | Muraina, Gwalior | 26^0^ 31’07.27” N | 78^0^00’10.49”E | Semi-arid |
| 47 | 57 | Bhind, Gwalior | 26^0^ 22’59.17” N | 78^0^ 18’24.45”E | Semi-arid |
| 48 | 58 | Jalalpur, Gwalior | 26^0^ 15’55.37” N | 78^0^ 09’47.16”E | Semi-arid |
| 49 | 59 | Bhitarwar, Gwalior | 25^0^ 05’53.71” N | 75^0^ 13’26.25”E | Semi-arid |
| 50 | 60 | Ramatpura, Gwalior | 25^0^ 13’24.91” N | 78^0^ 10’27.53”E | Semi-arid |
| 51 | 61 | Faizabad, UP | 26^0^ 17’00.57” N | 82^0^ 08’34.59”E | Humid subtropical |
| 52 | 62 | Ballia, Kakeri, | 25^0^ 42’42.59” N | 84^0^ 00’53.21”E | Humid subtropical |
| 53 | 63 | Ghazipur,UP | 25^0^ 51’50.91” N | 83^0^ 33’33.19”E | Humid subtropical |
| 54 | 64 | Chandauli, UP | 25^0^ 10’21.40” N | 83^0^ 17’35.39”E | Humid subtropical |
| 55 | 65 | , Ghazipur, UP | 25^0^ 51’43.70” N | 83^0^ 34’05.64”E | Humid subtropical |
| 56 | 66 | Pusa, samastipur | 25^0^ 59’03.99” N | 85^0^ 39’32.57”E | Humid subtropical |
| 57 | 67 | Pratapur Nigoha, Kanpur | 26^0^ 09’24.78” N | 86^0^ 04’37.88”E | Humid subtropical |
| 58 | 68 | Katra Ghanshyampur, Kanpur | 26^0^ 38’42.69” N | 80^0^ 12’49.29”E | Humid subtropical |
| 59 | 69 | Dharmayalpur, Kanpur | 26^0^ 31’28.70” N | 80^0^ 14’54.19”E | Humid subtropical |
| 60 | 70 | Katra, Kanpur city | 26^0^ 26’54.03” N | 80^0^ 08’31.47”E | Humid subtropical |
| 61 | 71 | Ganagapur, Kanpur | 26^0^ 31’42.27” N | 80^0^ 03’07.29”E | Humid subtropical |
| 62 | 72 | Anoopur, Ramabynagar | 26^0^ 32’27.75” N | 80^0^ 03’02.61”E | Humid subtropical |
| 63 | 73 | Dharmangalpur, Kanpur | 26^0^ 26’37.96” N | 80^0^ 08’44.34”E | Humid subtropical |
| 64 | 74 | Yugrajpur, Ramabynagar | 26^0^ 31’39.20” N | 79^0^ 49’53.77”E | Humid subtropical |
| 65 | 75 | Elavenchary Vattekkad, Palakkad | 10^0^ 36’23.02” N | 76^0^ 38’59.26”E | Tropical wet |
| 66 | 76 | Alappad(TCR) Thrissur | 10^0^45’52.8” N | 76^0^ 40’23.85”E | Tropical wet |
| 67 | 77 | Vallongi, palakkad | 10^0^26’24.09” N | 76^0^ 10’06.26”E | Tropical wet |
| 68 | 78 | Melapalur, Ariyalur | 11^0^02’06.76” N | 79^0^ 03’05.46”E | Tropical wet and dry |
| 69 | 79 | Jabalpur | 23^0^13’48.0” N | 79^0^58’18.1”E | Humid subtropical |
| 70 | 80 | Jabalpur | 23^0^13’47.9” N | 79^0^58’17.6”E | Humid subtropical |
| 71 | 81 | Jabalpur | 23^0^13’49.5” N | 79^0^58’16.8”E | Humid subtropical |
| 72 | 82 | Jabalpur | 23^0^13’48.1” N | 79^0^58’19.5”E | Humid subtropical |
| 73 | 83 | Panagar | 23^0^16’02.4” N | 79^0^59’55.6”E | Humid subtropical |
| 74 | 84 | Panagar | 23^0^16’06.5” N | 79^0^59’53.0”E | Humid subtropical |
| 75 | 85 | Mehgawa | 23^0^18’60.0” N | 80^0^01’50.9”E | Humid subtropical |
| 76 | 86 | Raipur | 21^0^15’56.10”N | 81 ^0^40’57.04”E | Humid subtropical |

* Collections already available at the Directorate
